# Ferroptosis as a potential therapeutic target for obesity-related metabolic diseases

**DOI:** 10.3389/fphar.2025.1581632

**Published:** 2025-07-04

**Authors:** Chao-Dong Huang, Tao Luo, Hua Zhang, Lin-Hui Cheng, Shu-Hong Peng, Xiao-Wei Zhou, Lan Cao, Fang-You Chen, Jun-Wei He, Chen Chen, Chang-Hua Zhang, Yu-Ai Zhang, Ling-Yun Zhong

**Affiliations:** ^1^ College of Pharmacy, Jiangxi University of Chinese Medicine, Nanchang, Jiangxi, China; ^2^ Blood Purification Center, The First Affiliated Hospital of Nanchang University, Nanchang, China; ^3^ Department of Radiology, The Third Affiliated Hospital, Jiangxi Medical College, Nanchang University, Nanchang, China; ^4^ Department of Radiology, The First Hospital of Nanchang, Nanchang, China; ^5^ Nanchang Research Institute, Sun Yat-sen University, Nanchang, Jiangxi, China; ^6^ Research center for differentiation and development of TCM basic theories, Jiangxi University of Chinese Medicine, Nanchang, Jiangxi, China; ^7^ Changzhou West Taihu Science and Technology Industrial Park Management Committee, Changzhou, Jiangshu, China; ^8^ School of Biomedical Sciences, University of Queensland, Brisbane, QLD, Australia; ^9^ School of Pharmacy, Fudan University, Shanghai, China

**Keywords:** ferroptosis, obesity-related metabolic diseases, occurrence and regulation of ferroptosis, pharmacotherapy strategy, potential therapeutic targets

## Abstract

Obesity represents one of the major public health issues threatening the global health and promoting chronic metabolic disorders, including type 2 diabetes, insulin resistance (IR), hyperlipidemia, hypertension, polycystic ovary syndrome, metabolic-associated fatty liver disease (MAFLD), and others. Ferroptosis, a novel form of cell death, is a programmed cell death induced by iron-dependent lipid peroxidation. It is characterized by excessive iron accumulation and unregulated lipid peroxidation. The activity of ferroptosis is modulated by multiple factors such as iron, reactive oxygen species, and over 98 unsaturated fatty acids. Mounting evidence indicates that ferroptosis plays a crucial role in obesity-related chronic metabolic diseases like type 2 diabetes, IR, hyperlipidemia, hypertension, polycystic ovary syndrome, and MAFLD. Clarifying the molecular mechanism of ferroptosis may discover potential therapeutic targets for the treatment of these diseases. This article comprehensively reviews the role, pathogenesis, prevention, treatment strategies, current research gaps and future development directions of ferroptosis in obesity-related chronic metabolic diseases have been thoroughly discussed, and novel perspectives for the future treatment and research of ferroptosis in these diseases carefully provided. It points out directions for basic research on ferroptosis, raises urgent needs for developing precise intervention strategies, and provides new insights into the treatment and study of obesity-related chronic metabolic diseases in the future.

## 1 Introduction

Obesity is a chronic medical condition characterized by excessive fat accumulation, contributing to systemic metabolic dysregulation and diverse health complications. This condition has been recognized as a chronic disease by leading medical associations such as the American Association of Clinical Endocrinologists (AACE) and the American Endocrine Society (ACE). The World Health Organization (WHO) classifies obesity as a systemic disease due to its widespread impact on bodily functions and overall health (Zhu S. et al., 2021). The 2017 WHO Global Disease Report revealed alarming statistics about the worldwide obesity epidemic. The report estimated that there were a staggering 107.7 million children and 603.7 million adults globally who were classified as obese in 2015. Shockingly, this translates to an overall obesity rate of 5.0% among children and 12.0% among adults (He et al., 2023). Obesity is a systemic disorder that gives rise to metabolic abnormalities in multiple systems and can result in a series of chronic metabolic diseases, such as type 2 diabetes, IR, hyperlipidemia, hypertension, polycystic ovary syndrome and MAFLD (Jia et al., 2021; Llovet et al., 2023). Mounting evidence indicates that ferroptosis plays a significant role in obesity-related chronic metabolic diseases.

Iron-dependent necroptosis, also known as ferroptosis, was formally proposed and named in 2012, when a small molecule compound-erastin triggered a unique iron-dependent non-apoptotic form of cell death (Du et al., 2022; Kim et al., 2020). Erastin inhibited the transport of cysteine into cells, leading to a decrease in glutathione (GSH) and the inactivation of glutathione peroxidase 4 (GPX4), ultimately inducing cell death. Ferroptosis plays a significant role in obesity and obese-related complications. The activity of ferroptosis is regulated by various factors, including iron, reactive oxygen species and polyunsaturated fatty acids (Wang et al., 2022; Hara et al., 2020).

In this review, the role, pathogenesis, prevention, treatment strategies, current research gaps and future development directions of ferroptosis in obesity-related chronic metabolic diseases have been thoroughly discussed, and novel perspectives for the future treatment and research of ferroptosis in these diseases carefully provided. While ferroptosis mechanisms have been extensively reviewed (Dixon and Olzmann, 2024), this article uniquely focuses on obesity-driven metabolic dysregulation, emphasizing three novel directions: (1) tissue-specific iron overload in adipose tissue and the hepatic system; (2) upregulation of hepcidin mediated by adipokines as a key amplifier of ferroptosis; (3) the dual regulatory role of Traditional Chinese Medicine (TCM) compounds in the modulation of ferroptosis. It points out directions for basic research on ferroptosis, raises urgent needs for developing precise intervention strategies, and provides new insights into the treatment and study of obesity-related chronic metabolic diseases in the future.

## 2 Occurrence and regulation of ferroptosis

### 2.1 Occurrence of ferroptosis

**FIGURE 1:**
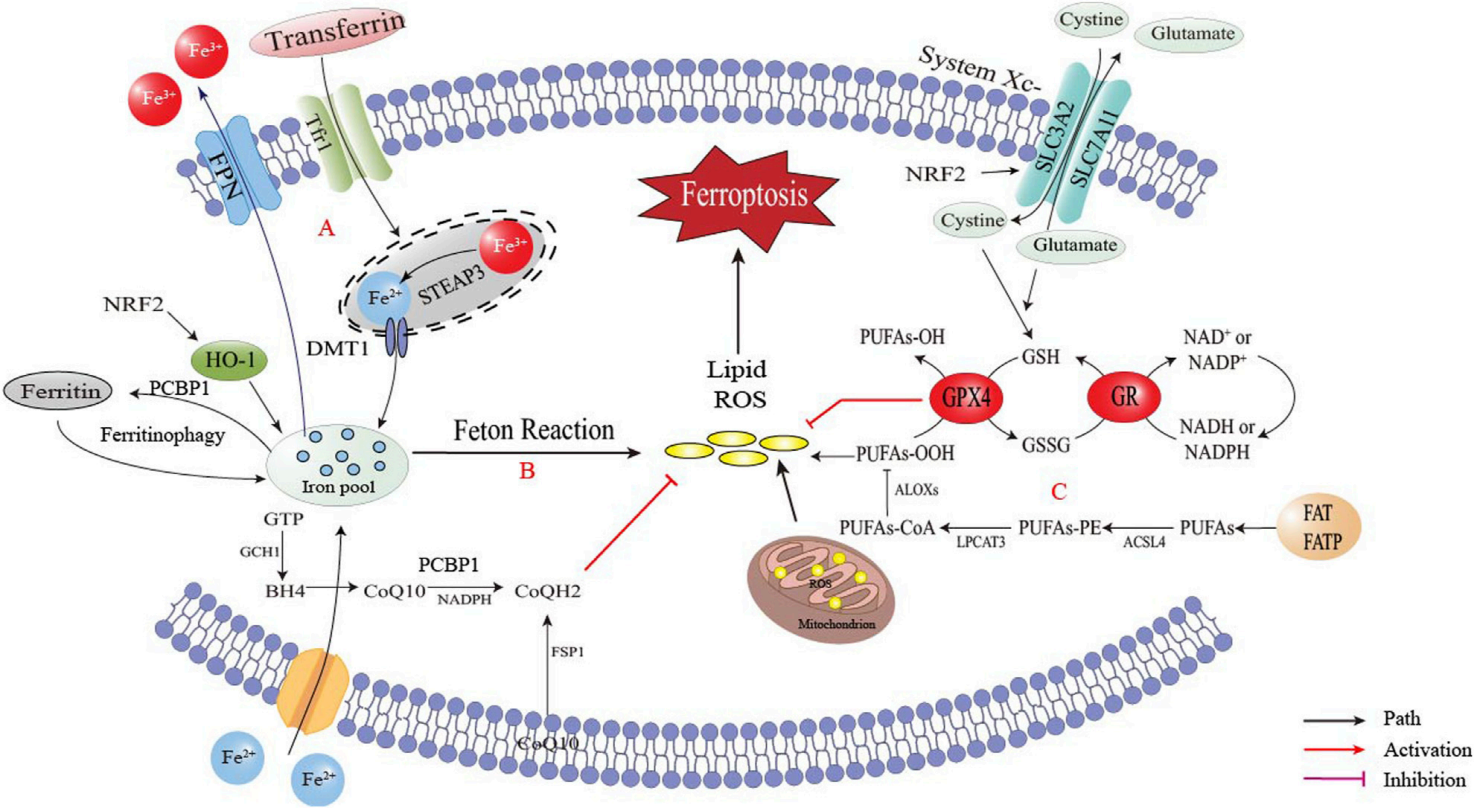
The occurrence of ferroptosis. Disorders of intracellular iron metabolism led to iron accumulation, and iron in the labile iron pool (LIP) induced Fenton reaction and generate ROS. ROS led to lipid peroxidation, the destruction of membrane structure in cells and eventually caused cell death. **(A)** Iron accumulation via TfR1 (transferrin receptor 1)/FPN (ferroportin) imbalance; **(B)** Generation of reactive oxygen species (ROS) via Fenton reaction and mitochondrial dysfunction; **(C)** ACSL4 (acyl-CoA synthetase long-chain family member 4)/LOX (lipoxygenase)-mediated lipid peroxidation cascade.

Ferroptosis is a type of cell death that is characterized by the accumulation of lipid reactive oxygen species (ROS) induced by iron, which damages proteins, nucleic acids and lipids, leading to intracellular oxidative stress and ultimately cell death. It has distinct morphological, biochemical and molecular characteristics from other forms of cell death such as necrosis, apoptosis, exophthalmos and autophagy. Although iron is an essential nutrient for cell proliferation, excess iron produces catalytic Fe^2+^ *in vivo*, which causes the Fenton reaction leading to the production of hydroxyl radicals that attack lipids, peroxidase polyunsaturated fatty acid (PUFA) and cause ferroptosis of cells (Hara et al., 2020). Ferroptosis involves three key processes: free iron accumulation, oxidative stress and lipid oxidative damage that induces cell membrane degeneration, as shown in Figure 1.

#### 2.1.1 Free iron accumulation

The occurrence of ferroptosis requires the presence of iron ions, and various factors can influence cell ferroptosis by regulating the transport and metabolism of iron ions. Iron is a crucial trace element in the human body, essential for most cellular biological processes, such as DNA replication, mitochondrial respiration and cell signaling (Pan et al., 2021) (Menon et al., 2022) (Capelletti et al., 2020). Therefore, disorders in iron metabolism, particularly iron overload, may significantly impact the development of various metabolic diseases (Hara et al., 2020). Iron in cells and serum maintains iron homeostasis in the body, and both intracellular iron import and export can impact the sensitivity of cells to ferroptosis (Lee et al., 2021). The import of extracellular iron into the cell is mainly regulated by transferrin (Trf) and transferrin receptor 1 (Tfr1), with Trf being a metal-binding protein synthesized primarily in the liver. Serum iron is mainly bound to Trf, known as transferrin-bound iron (TBI) (Galy et al., 2024). As illustrated in Figure 1A, non-transferrin-bound iron (NTBI) accumulates when serum iron levels surpass the buffering capacity of transferrin, resulting in toxicity that can be cleared by the liver. Iron overload in obesity is closely linked to dysregulated hepcidin and transferrin pathways, leading to intracellular iron accumulation.

#### 2.1.2 Unstable iron pool (LIP) and autophagy by ferritin

Iron is either used functionally or stored in ferritin or the labile iron pool (LIP). If there is an excess, it may be removed from the cell through ferroportin (Hara et al., 2020). Changes in LIP levels affect a cell’s sensitivity to ferroptosis. Ferritin, which is the primary form of iron storage, plays a role in regulating ferroptosis (Chen et al., 2020a). As illustrated in Figure 1B, ferroptosis may be initiated by reduced ferritin expression or increased LIP (Hou et al., 2016). Autophagy may also promote ferroptosis by breaking down ferritin, which stores the iron that the cell has absorbed. On the other hand, inhibiting autophagic degradation of ferritin increases LIP content and makes cells more resistant to ferroptosis (Gao et al., 2016). Mitochondria, which also store iron, increase LIP content during autophagy. Inhibiting mitochondrial metabolism may promote autophagy, increase LIP availability and make cells more susceptible to ferroptosis (Wang et al., 2022).

#### 2.1.3 Poly (rC) binding protein 1 (PCBP1) and heme oxygenase-1 (HO-1)

PCBP1 functions as a cytosolic iron chaperone that moves iron to ferritin and other non-heme iron requiring proteins (Galy et al., 2024). Research conducted *in vitro* has revealed that cytoplasmic iron pools primarily exist in the form of PCBP1-Fe-GSH complexes and that PCBP1 governs Fe-GSH reactivity as well as the migration of unstable iron pools (Patel et al., 2019). HO-1, a significant antioxidant enzyme, mainly transforms heme to ferrous iron, carbon monoxide and biliverdin. HO-1 promotes ferroptosis, metabolize heme and release ferrous iron (Fang et al., 2019). Previous investigations discovered that HO-1 accelerated erastin-induced ferroptosis. A recent study reported that an abnormal activation of HO-1 was stimulated by the nuclear factor erythroid 2-related factor 2 (NRF2)-SLC7A11-HO-1 signaling pathway, which led to ferroptosis of retinal pigment epithelial cells (Tang et al., 2021). In contrast, other studies stated that the knockdown of HO-1 promoted ferroptosis. NRF2 mitigated ferroptosis by upregulating recombinant solute carrier family 7, member 11 (SLC7A11) and HO-1. As a solution to the above problems, it was suggested (Wang et al., 2022) that HO-1 was an enzyme that catalyzes the degradation of heme to ferrous iron, carbon monoxide and biliverdin. Excessive activity of HO-1 raised LIP and subsequently caused ferroptosis. However, based on its antioxidant activity, a modest upregulation of HO-1 may protect cells (Kim et al., 2020).

#### 2.1.4 ROS metabolism

ROS are vital indicators of ferroptosis and are made up of partially reduced oxygen-containing molecules like peroxide (H_2_O_2_) and superoxide (O^2−^), which originate from various sources such as singlet oxygen (1O_2_) and free radicals (HO·, RO·, NO· and NO_2_·) (Lin et al., 2018; Conrad and Pratt, 2019). GPX4 is the central downstream regulator of ferroptosis, as it reduces toxic phospholipid hydroperoxides to nontoxic phosphatidyls, maintaining cellular redox balance with the help of GSH. Cystathionine-β-synthase activation, under the circumstance of the presence of ROS, promotes the trans-sulfur pathway, converts methionine into cysteine, synthesizes GSH and ultimately protects cells from ROS damage (Kim et al., 2020). As illustrated in Figure 1B, the Fenton reaction and the mitochondrial electron transport chain are major sources of ROS during ferroptosis (Capelletti et al., 2020).

#### 2.1.5 Lipid peroxidation and membrane structural changes

As illustrated in Figure 1C, lipid peroxidation is a hallmark of ferroptosis, driven by Acyl-CoA synthetase long-chain family member 4 (ACSL4) and lys phosphatidylcholine acyltransferase 3 (LPCAT3) mediated incorporation of PUFAs into membrane phospholipids (PL). These PUFAs are highly susceptible to oxidation by lipoxygenases (LOXs), leading to membrane destabilization (Yang et al., 2016; Carlson et al., 2016; Wu et al., 2021). Mitochondrial dysfunction synergizes with lipid peroxidation, as evidenced by cristae loss and outer membrane rupture, which further amplifies oxidative stress (Riegman et al., 2020; Jiang et al., 2021; Wang et al., 2020). GPX4 and ferroptosis suppressor protein 1 (FSP1) are critical regulators that counteract lipid peroxidation by reducing lipid hydroperoxides or regenerating antioxidants like coenzyme Q10(CoQ10) (Yang et al., 2014; Bersuker et al., 2019; Doll et al., 2017).

#### 2.1.6 Mitochondrial dysfunction in ferroptosis

Mitochondria are central to ferroptosis due to their role in ROS generation and iron metabolism. In obesity, mitochondrial cristae loss and impaired electron transport chains exacerbate lipid peroxidation (Wang et al., 2020). Additionally, mitochondrial GPX4 depletion sensitizes cells to ferroptosis, as seen in diabetic cardiomyopathy models (Fang et al., 2019).

### 2.2 Regulation of ferroptosis

**FIGURE 2:**
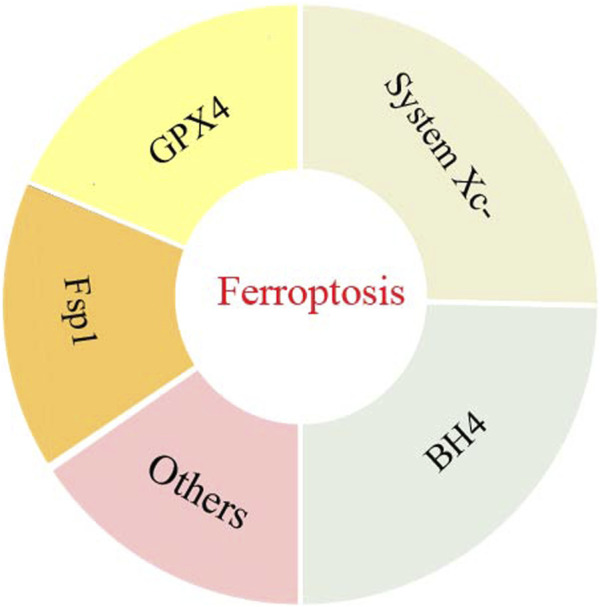
The key systems involved in the regulation of ferroptosis lipid peroxidation in obesity-related chronic metabolic diseases.

The complex molecular mechanism behind ferroptosis involves precise regulation of oxidative stress, lipid peroxidation and iron homeostasis in cells. Among these regulatory pathways, lipid peroxidation plays a critical role in the ferroptosis network. An imbalance between factors that promote and antagonize lipid peroxidation can increase lipid peroxidation and contribute to the onset of ferroptosis (Lei et al., 2022). To regulate lipid peroxidation, the synthesis of polyunsaturated fatty acyl moieties in phospholipids (PUFA-PL), LIP, mitochondrial ROS synthesis and Xc-system activity may promote lipid peroxidation. At the same time, the activities of cyst(e)ine-GSH-GPX4 axis, NAD (P) H-FSP1-CoQ10 axis, GTP cyclohydrolase 1 (GCH1)- tetrahydrobiopterin (BH4)- dihydrofolate reductase (DHFR) axis and DHOOH-CoQH2 system all inhibit lipid peroxidation. As illustrated in Figure 2, this section has briefly overviewed these key systems that are involved in the regulation of ferroptosis lipid peroxidation in obesity-related chronic metabolic diseases.

#### 2.2.1 Cystine/glutamate reverse transport system (system Xc-)

The system Xc-plays a crucial role in regulating ferroptosis and inhibition of its function may prevent this process. This system facilitates the exchange of cystine and glutamate in a Na^+^-dependent manner, with a 1:1 stoichiometric ratio of cystine import and glutamate export. It is composed of a disulfide-linked heterodimer, consisting of a light chain subunit recombinant solute carrier family 7, member 11 (SLC7A11/xCT) and a heavy chain subunit (CD98hc, recombinant solute carrier family 3, Member 2-SLC3A2) (Koppula et al., 2018). Additionally, there are Na^+^-independent anti-transporters that import cystine and export glutamate in an ATP-dependent manner. Once cystine is internalized into the cells, it will be rapidly reduced to cysteine involved in GSH synthesis (Koppula et al., 2021). Inhibiting system Xc-reduces GSH synthesis by blocking cystine absorption may impair the function of GPX4, leading to the reduction of cellular antioxidant capacity and the accumulation of lipid ROS, and causing ferroptosis ultimately (Lei et al., 2022).

#### 2.2.2 Cyst(e)ine-GSH-GPX4

The GPX4 gene plays a crucial role in scavenging lipid peroxidation, the negative effects of GPX4 knockout or pharmacological inhibition can prove it, which results in uncontrolled lipid peroxidation (Yang et al., 2014). The function of GPX4 converts GSH into oxidized glutathione (GSSG) and reduces harmful lipid peroxides to harmless lipid alcohols. On the other hand, glutathione reductase reduces GSSG to GSH through a nicotinamide adenine dinucleotide phosphate (NADPH)-dependent process. RSL3 is a potent inhibitor of GPX4, it can trigger lipid peroxidation and ROS production when combined with GPX4, leading to ferroptosis (Zhou et al., 2020). However, RSL3 inhibitors may reverse this effect (Shintoku et al., 2017). In the research of ferroptosis-inducing compounds, ferroptosis-inducing compounds (FINs) were discovered, including diphenyl iodonium iodide (DPI) 7/10/12/13/17/18/19 and FIN56, which directly inhibited GPX4 activity without affecting GSH levels (Liang D. et al., 2022). Nevertheless, GPX4 inactivation caused by GSH depletion promoted ferroptosis by increasing ROS levels in lipid peroxidation (Hara et al., 2020).

#### 2.2.3 NAD (P) H-FSP1-CoQ10

The FSP1-CoQ10-NAD(P)H complex is a self-regulating system near the cell membrane and works with GPX4 and GSH to prevent phospholipid peroxidation (PLPO) and ferroptosis (Doll et al., 2019). FSP1, a member of the NDH-2 family, uses nicotinamide adenine dinucleotide (NADH) to convert CoQ into ubiquinol, then captures lipid peroxides (Yan et al., 2021). Recent research indicated that FSP1 played a crucial role in preventing ferroptosis by providing CoQ and GPX4. The regulation of FSP1 involves several factors, including p53, miR-214, cAMP response element binding protein, NRF2, murine double minute 2 (MDM2)-MDMX compound and its transcription factor PPARa (Nguyen et al., 2020; Venkatesh et al., 2020).

#### 2.2.4 GCH1-BH4-DHFR

BH4 not only acts as a cofactor for nitric oxide synthases but also promotes CoQ10 biosynthesis by providing 4-hydroxybenzoate precursors, thereby enhancing antioxidant capacity and mitigating ferroptosis (Kraft et al., 2020). GCH1 reduces lipid peroxidases in a GSH-independent manner, generates 6 (R)-L-red-5, 6, 7, 8-BH4 to sequester lipid peroxidases and offset the effects of iron ions (Yan et al., 2021). The regeneration of BH4 needs the involvement of DHFR (Chen et al., 2022). The combination of Dihydrofolate reductase blockers and GPX4 inhibition can promote ferroptosis. However, the exact function of the GCH1-BH4-DHFR axis in this process remains to be clarified (Wang et al., 2022).

#### 2.2.5 Other systems

It was discovered that the heat shock factor 1 (HSF1)- HSP-binding factor 1 (HSPB1) signaling pathway blocked erastin-induced ferroptosis. This was achieved by reducing iron-mediated lipid reactive oxygen species through protein kinase C-mediated phosphorylation of HSPB1 (Sun et al., 2015a). The p62-Keap1-NRF2 signaling pathway also combated lipid peroxidation by pre-regulating multiple genes of iron and ROS metabolism. These genes include quinone oxidoreductase 1 (NQO1), HO-1 and ferritin heavy chain 1 (FTH1), all of them may inhibit ferroptosis in liver cancer cells (Sun et al., 2016). Some recent studies have shown that the AMP-activated Protein Kinase (AMPK)-ACC and NF2 (also known as merlin)-YAP signaling pathways also inhibit ferroptosis. Such inhibition is accomplished through modulating PUFAS energy stress-mediated AMPK activation, as part of environment-dependent mitochondria-independent mechanisms (Lee et al., 2020; Wang, 2022). The p53 is a classical tumor suppressor that can prevent ferroptosis by inhibiting cysteine uptake and reducing the level of GSH. This is achieved by reducing SLC7A1 expression and then inactivating the system XC. However, in certain circumstances, the p53 may actually inhibit ferroptosis. The p53 limited erastin-induced ferroptosis by promoting the nuclear accumulation of dipeptidyl peptidase 4 (DPP4) and increasing the expression of SLC7A11 in human colorectal cancer (Wu et al., 2021; Xie et al., 2017). Brca1-associated protein 1 (BAP1) is an H2A deubiquitinase that induces ferroptosis of cancer cells by inhibiting SLC7A11. Additionally, nuclear factor erythroid 2-related factor 2 (NFR2) is a regulator against oxidative stress that activates SLC7A11 by binding to antioxidant response elements (Jia et al., 2021).

### 2.3 The physiological and biochemical functions of ferroptosis

#### 2.3.1 In terms of morphology

When cell apoptosis, the cell membrane does not rupture, and typical apoptotic bodies appear within the cytoplasm. When cell necrosis, the cell swells; the nucleus condenses, ruptures and dissolves; the chromatin staining becomes pale and filamentous; and organelles swell or rupture. Ferroptosis, however, lacks typical apoptotic and necrotic morphological features, mainly characterized by cell membrane rupture, vesiculation, shrinkage of mitochondria, increase in density of membrane, decrease in cristae and lack of condensation of chromatin in the nucleus, etc (Gautheron et al., 2020).

#### 2.3.2 In terms of biochemical characteristics

The apoptotic process mainly relies on cysteinyl aspartate-specific proteinase (Caspase), which is a protein enzyme containing cysteine. When apoptosis, the cytoplasmic Ca^2+^ and pH levels rise, nuclear DNA is fragmented by activated endonuclease, cell membrane phosphatidylserine appears ectropion, the mitochondrial membrane potential decreases and permeability increases. Cell necrosis causes a severe inflammatory response in the local area, which is related to various signaling pathways, including receptor interacting protein kinase 3 (RIPK3). However, when ferroptosis, Fe^2+^ in the cell starts gathering; lipid peroxidation level rises remarkably; ROS increases; the cysteine intake of cell decreases; GSH depletes and pro-inflammatory mediators such as arachidonic acid (AA) releases (Dixon et al., 2012). The essence of ferroptosis is the accumulation of Fe^2+^ and the disruption of cellular oxidative-reduction metabolism-a decrease in the cell’s antioxidant capacity. Because the products of ROS and lipid peroxidation in the cell cannot be restored, these products abundantly accumulate and ultimately lead to cell death. Both cell apoptosis and necrosis cells can be isolated to ferroptosis, and small molecules that inhibit cell apoptosis and necrosis have no effect on ferroptosis (Cui et al., 2017).

## 3 The role of ferroptosis in obesity and obesity-related metabolic diseases

**FIGURE 3:**
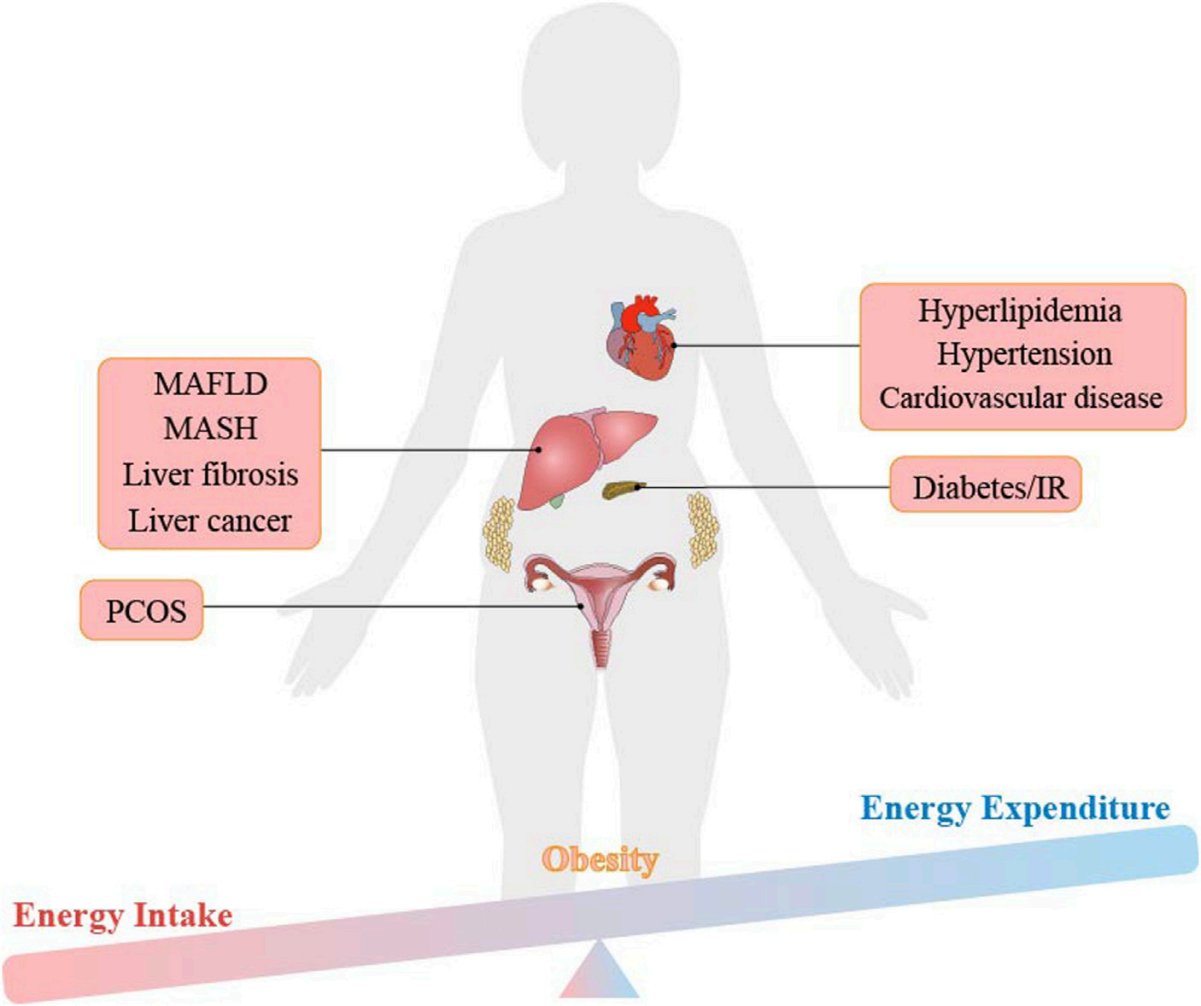
The imbalance between energy intake and energy expenditure leads to obesity and obesity-related metabolic diseases.

Ferroptosis is related to the development of obesity. When we intake too much nutrient, it will lead to the storage of fat in our white adipose tissue (WAT), which ultimately leads to weight gain and obesity, as shown in Figure 3 (He et al., 2023; Zhang et al., 2022). Iron metabolism is dysregulated in obese individuals, despite intaking enough iron in their diet, causing lower iron levels than that of non-obese people (Frelut et al., 2018). A meta-analysis of 21 studies conducted on overweight and non-obese individuals found a significant correlation between obesity and iron deficiency (Puri et al., 2017; Zhang et al., 2021). Iron loss was also found to play a role in the degradation of damaged mitochondria (mitophagy) (Sies and Jones, 2020; NCD Risk Factor Collaboration NCD-RisC, 2024). In summary, increased levels of hepcidin and decreased levels of transferrin in obese individuals prevent excess intracellular iron from being excretion and instead keep it stored in the cell as free iron. These free iron increases the risk of ferroptosis and further aggravates the inflammatory response, long-term chronic inflammatory response may lead to the occurrence of various metabolic diseases (Wang et al., 2022; Puri et al., 2017).

Metabolic diseases such as type 2 diabetes, IR, hyperlipidemia, MAFLD, hypertension and atherosclerosis are all directly related to the occurrence of obesity, as shown in Figure 3 (Qiu et al., 2022). In addition, studies have shown that these obesity-related metabolic diseases are accompanied by changes in iron content in the body. Dietary iron is transported in the small intestine or ileum, a process mediated by the divalent metal transporter 1 (DMT1) of iron transporter (Gulec et al., 2014). Excess iron in the body is stored in the form of ferritin, mainly in the liver, spleen, bone marrow and small intestinal mucosa. Usually, the transferrin-ferritin axis is responsible for maintaining iron homeostasis in the body. Studies have proven that reducing plasma ferritin can improve. MAFLD in obese patients, indicating that considering iron content is crucial in the treatment of obesity-related metabolic diseases (Moore Heslin et al., 2021). In this review, the mechanism of ferroptosis is carefully discussed in obesity and related metabolic diseases, suggesting new viewpoints and evidence of ferroptosis as the therapeutic target.

### 3.1 Ferroptosis and diabetes and its complications

Diabetes is a lifelong metabolic disease with multiple etiologies, characterized by chronic hyperglycemia (Alberti and Zimmet, 2013). Long-term metabolic disorders and continuous hyperglycemia can gradually aggravate the damage to the nervous system and systemic tissues and organs such as the cardiovascular and kidney, resulting in various complications (Chowdhury et al., 2015). According to the statistics from the International Diabetes Federation in 2019, the incidence and mortality of diabetes increased every year, threatening people’s physical and mental health (Saeedi et al., 2019; Saeedi et al., 2020). Therefore, in-depth exploration of the pathogenesis of diabetes and its complications and emerging treatment opportunities is the goal of global public health (He et al., 2022). Ferroptosis may open a new door in our understanding of diabetes and its complications. A large number of studies have shown that ferroptosis plays a crucial role in the occurrence and development of diabetes and its complications.

In obesity, chronic inflammation and adipokine dysregulation (e.g., leptin resistance) induce pancreatic β-cell iron overload through hepcidin-mediated ferroportin (FPN) suppression (Zhang et al., 2022). Excess intracellular Fe^2+^ fuels Fenton reactions, generating mitochondrial ROS (mtROS) that damage β-cell insulin secretory machinery. Adipose-derived TNF-α further exacerbates this by downregulating GPX4 via NF-κB, as shown in high-fat diet (HFD) mouse models (He et al., 2022). Notably, nuclear receptor coactivator 4 (NCOA4)-mediated ferritinophagy in β-cells amplifies LIP levels, creating a vicious cycle of oxidative stress and ferroptosis (Miao et al., 2023). Understanding the regulatory mechanism of iron metabolism and ferroptosis in pancreatic β-cells is helpful for the prevention of type 2 diabetes and the development of new therapeutic targets. The main active ingredient of Cryptochlorogenic Acid (CCA) in mulberry leaf extract may inhibit ferroptosis in diabetic rat models by activating the cystine/glutamate transporter system (XC-)/GPX4/NRF2, inhibit pathways such as NCOA4, and produce a good hypoglycemic effect (Zhou, 2020), suggesting ferroptosis as a therapeutic target of obesity-related metabolic diseases.

While ferroptosis contributes to pancreatic β-cell dysfunction in diabetes, its role in diabetic complications such as nephropathy and retinopathy involves distinct molecular pathways. In diabetes, most complications often appear in the later stage, such as diabetic kidney disease (DKD), diabetic retinopathy, diabetic neuropathy, diabetic foot and cardiovascular diseases. Among these complications, diabetic kidney disease is a very serious complication of diabetes, occurring in 40% of diabetic patients (Hong et al., 2021). When it develops into end-stage renal disease, it may bring serious psychological and economic burdens to patients and the society (Wu and Chen, 2022). The regulation of ferroptosis may intervene and improve diabetic kidney disease. The extracts of Orthosiphon stamineus leaves contained many polyphenols and reduced fasting blood glucose (FBG) and inflammation in diabetic nephropathy mice and improved the state of kidney damage (Zheng et al., 2024). At the same time, Orthosiphon stamineus leaves may also upregulate the expression of GPX4 and FTH1 and downregulate the expression of ACSL4 and NCOA4, indicating that it can inhibit ferroptosis in the kidney and slow down the development of diabetic nephropathy. NRF2 is an important factor in the regulation of ferroptosis. Enhancing the expression of NRF2 factor in diabetic nephropathy may inhibit ferroptosis and alleviate or treat diabetic nephropathy (Ji et al., 2023; Lei et al., 2024).

Diabetic retinopathy (DR) is a common microvascular complication of diabetes and causes specific pathological changes in the fundus (Tey et al., 2019; Stitt, 2010). Approximately 85% of patients with diabetic retinopathy have a background of type 2 diabetes. In addition, the prolongation of the course of diabetes in patients increases the incidence of diabetic retinopathy. Controlling the progression of diabetic retinopathy becomes a critical goal for improving the quality of life of diabetic patients (Tabatabaei-Malazy et al., 2020; Singh et al., 2019). Adult retinal pigment epithelial cell line-19 (ARPE19) cells were treated *in vitro* with high concentration glucose (high glucose, HG) to form a diabetic retinopathy model (Zhu Z. et al., 2021). Reduction of circular RNA PSEN1 in the miR-200b-3p/cofilin-2 axis improved the ferroptosis of retinal pigment epithelial cells treated with high glucose, thereby delaying the progression of diabetic retinopathy. Diabetic cardiovascular disease is also a complication of diabetes. Diabetic patients often have symptoms such as coronary artery stenosis, spasm and even angina pectoris (Lejay et al., 2016). The reason is that long-term diabetes may cause excessive ROS in tissues and organs, leading to lipid peroxidation (LPO) and ferroptosis in the heart (Latunde-Dada, 2017). Rhapontigenin may inhibit mitochondrial-related ferroptosis through the PRDX2 - MFN2 - ACSL4 pathway and reduce diabetic heart microvascular injury (Chen et al., 2022). Other diabetic complications are also closely related to ferroptosis. Many studies have demonstrated the pathogenic role of ferroptosis in diabetes and its complications. Therefore, it may be of great significance for the pathophysiology of diabetes and its complications and the development of effective treatment methods in the future.

### 3.2 Ferroptosis and hyperlipidemia

Hyperlipidemia is a common metabolic disease, referring to the condition where the level of lipids (mainly including cholesterol, triglycerides, PL and free fatty acids, etc.) in plasma is higher than the normal range (Xie et al., 2016). Obesity-driven hyperlipidemia elevates circulating PUFAs (e.g., AA), which are incorporated into hepatocyte membranes via ACSL4/LPCAT3 (Yang et al., 2014). In the context of adipose tissue hypoxia, adipocyte-derived extracellular vehicles (EVs) deliver miR-27a-3p to hepatocytes, silencing FSP1 and disabling CoQ10-dependent antioxidant defenses (Zhang et al., 2020). This dual hit (lipid overload + antioxidant failure) primes hepatocytes for ACSL4/LOX-mediated lipid peroxidation, driving hepatic IR. In the research on treating hyperlipidemia, ferroptosis also plays a crucial role (Zhang et al., 2020). In vascular smooth muscle cells from hyperlipidemia mice pretreated with metformin, an increase in extracellular matrix protein periostin (POSTN) inhibited SLC7A11 expression by diminishing p53, thereby leading to a reduction in glutathione synthesis and causing ferroptosis (Ma W.-Q. et al., 2021).

### 3.3 Ferroptosis and hypertension/cardiovascular diseases

Hypertension is a metabolic disease characterized by continuously elevated arterial blood pressure in the systemic circulation. Obese people often have the increase of adipose tissue in the body, especially abdominal fat accumulation. This leads to an increase in peripheral vascular resistance because excessive adipose tissue releases some bioactive substances such as angiotensinogen and leptin. These substances may cause vasoconstriction and lead to an increase in blood pressure. Long-term hypertension causes cardiovascular and other diseases. Lifestyle improvement and drug treatment are needed to control blood pressure. Some recent studies have shown that ferroptosis plays an important role in the occurrence and development of hypertension. Lenvatinib, a multi-target tyrosine kinase inhibitor (TKI), inhibited the Yes-associated protein (YAP) in endothelial cells leading to ferroptosis of endothelial cells and subsequently vascular dysfunction and hypertension. It was suggested that the pathways regulating ferroptosis were closely related to the occurrence and development process of hypertension (Liang C. et al., 2022a). Hypertension is an important risk factor for cardiovascular diseases (CVD) (Yudan et al., 2020). Long-term hypertension may increase the burden on the heart, leading to myocardial hypertrophy and cardiac enlargement. The vascular endothelium may be damaged to promote atherosclerosis, coronary heart disease and stroke (Pistoia et al., 2015). Therefore, clearly understanding the pathophysiological basis behind the pathogenesis of cardiovascular diseases is of great significance for the prevention, early diagnosis and precise treatment of cardiovascular diseases (Choi et al., 2019). In recent years, strong evidence indicates that ferroptosis is involved in the development of cardiovascular diseases, including cardiomyopathy, heart failure, vascular injury, atherosclerosis (AS), cardiorenal syndrome (CRS), pulmonary arterial hypertension (PAH) and others (Zhaolin et al., 2018). Protopanaxatriol A may protect doxorubicin (dox)-induced myocardial injury and cardiac dysfunction by targeting the ACSL4/FTH1 axis-dependent ferroptosis, indicating that protopanaxatriol A has the potential to be developed as an anti-ferroptosis cardiac protective drug (Jingxuan et al., 2024). Maintaining iron homeostasis is crucial for normal cardiac function. Both iron deficiency and iron accumulation are associated with cardiovascular diseases through complex mechanisms. By crossing gene-deficient mice, mice with specific cardiomyocyte iron deficiency protein (ferritin H) were obtained (Xuexian et al., 2020). Administration of ferroptosis inhibitors improved symptoms such as heart injury and hypertrophic cardiomyopathy in the mouse model, indicating that ferritin plays a major role in preventing cardiac ferroptosis and subsequent heart failure, further providing evidence for ferroptosis as a new target of the prevention and treatment of heart diseases. Many studies also showed that ferroptosis plays an important role in cardiovascular diseases. For example, Micheliolide (MCL) inhibited macrophage ferroptosis by targeting the interaction of KEAP1/NRF2, thereby reduced atherosclerosis (Luo et al., 2023). Inhibiting glutaminolysis (an important component of ferroptosis) reduced ischemia-reperfusion-induced cardiac injury (Gao et al., 2015). The 15-lipoxygenase (Alox15) and 15-hydroperoxyeicosatetraenoic acid (15-HpETE) aggravated myocardial ischemia-reperfusion injury by promoting cardiomyocyte ferroptosis (Cai et al., 2023).

### 3.4 Ferroptosis and polycystic ovary syndrome

Polycystic ovary syndrome (PCOS) is a complex metabolic disease. Main pathological features are ovulation disorders, accompanied by clinical manifestations such as hyperandrogenism and endocrine hormone disorders (Adamska et al., 2020). The incidence of PCOS in Chinese women of childbearing age is 4%–12%, accounting for about 10% of gynecological diseases. Moreover, the incidence of long-term complications such as endometrial cancer and cardiovascular diseases increase significantly, seriously affecting women’s physical and mental health. Obese women with PCOS exhibit elevated adipose IL-6, which induces ovarian granulosa cell ferroptosis via JAK2/STAT3-mediated HO-1 activation (Hoeger et al., 2021). HO-1 degrades heme to release Fe^2+^, while IL-6 concurrently downregulates ovarian FTH1, depleting iron storage capacity. This iron-redox imbalance disrupts follicular development, as evidenced by GPX4-knockout mouse models showing accelerated ovarian failure (Assou et al., 2010). The human ovarian GC tumor cell line (KGN cells) was used to study the function and regulatory mechanism of granulosa cells (Tan et al., 2022). The overexpression of miR-93-5p in KGN cells promoted the occurrence of ferroptosis. The miR-93-5p promoted apoptosis and ferroptosis of GC cells by regulating the NF-kB signaling pathway. Through gene differential expression analysis, five genes, NOX1, ACVR1B, PHF21A, FTL and GALNT14, were identified from ten differentially expressed ferroptosis-related genes (Lin et al., 2023). These five genes may be related to the pathogenesis of PCOS and may be also used for clinical diagnosis and treatment of PCOS.

### 3.5 Ferroptosis and obesity-related liver disease

Obesity is a major risk factor for MAFLD as it leads to excessive synthesis of triglyceride of the liver due to an excess of circulating lipids. The liver may not be able to release it into circulation, leading to the accumulation of triglyceride in hepatocytes (parenchymal cells) in the liver. MAFLD may develop into chronic liver inflammation known as metabolic-associated steatohepatitis (MASH), which further may develop into liver fibrosis or hepatocirrhosis with severe complications, including liver failure and hepatocellular carcinoma (HCC) (Huby and Gautier, 2022; Loomba et al., 2021). The global prevalence of MAFLD is increasing and affects approximately 25% of the world’s population (Araújo et al., 2018). Both metabolically healthy and unhealthy obesity patients are associated with the occurrence and development of MAFLD, along with an increase in the prevalence of metabolic syndrome. Characterized by abnormal accumulation of lipid in the cytoplasm of hepatocytes, hepatic steatosis is the main feature of MAFLD, which is detectable by histology and non-invasive imaging (Lonardo et al., 2020; Yip et al., 2022). Other chronic liver diseases often coexist with hepatic steatosis, and there is plenty of data to support the idea that a low-fat diet can treat liver disease (Yki-Järvinen et al., 2021).

**FIGURE 4:**
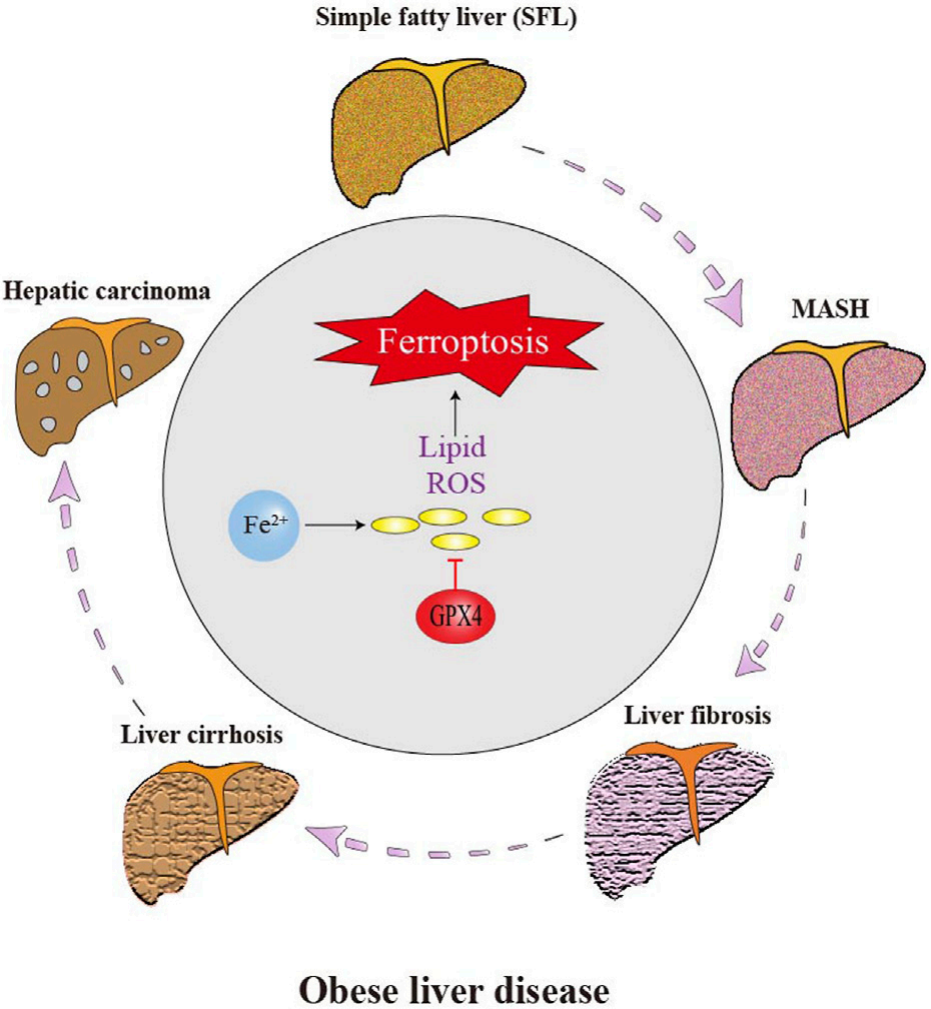
Effect of ferroptosis on the progression of obese liver disease. Liver cells are the main place of iron storage in the body and greatly affected by iron overload. The occurrence of ferroptosis greatly promotes the progression of obese-related fatty liver disease. Ferroptosis of hepatic stellate cell (HSC) and other cells aggravates liver inflammation, fibrosis and eventually HCC.

Figure 4 illustrates several types of fatty liver diseases caused by obesity, including MAFLD, MASH, non-alcoholic fatty liver cirrhosis and fibrosis. These conditions have a high probability to develop into liver cancer and are also related to cardiovascular disease and diabetes (Schwabe et al., 2020). Liver is the primary part for storing iron in the body, and if the iron level in liver tissue exceeds 13–15 mg/g, the risk of cirrhosis increases (Piperno et al., 2020). Iron overload in liver disease can be mainly attributed to two aspects: increased absorption in the intestines due to iron-mediated cell damage and excessive iron storage (Ma W. et al., 2021). The body strictly regulates iron levels through transferrin and ferritin. A deficiency in liver transferrin can lead to dyserythropoiesis, an increase in non-transferrin-bound iron content in the serum, and eventually, chronic iron deposition and liver damage (Gaschler and Stockwell, 2017). The correlation between ferroptosis and inflammation is well-established in various types of organ damage and degeneration (Deng et al., 2023). Evidence indicates that ferroptosis participates in the development of several liver diseases, including HCC, fibrosis, MASH, hepatic I/R injury and liver failure (Kim et al., 2020).

#### 3.5.1 Ferroptosis and metabolic-associated fatty liver disease (MAFLD)

The pathogenesis of MAFLD follows a “two-hit” model, where hepatic steatosis (first hit) primes the liver for ferroptosis-driven injury through dual mechanisms of iron dysmetabolism and redox imbalance (Gao et al., 2021). IR in obesity promotes lipolysis in adipose tissue, releasing free fatty acids (FFAs) that upregulate ACSL4 in hepatocytes, thereby enriching membranes with PUFAs susceptible to peroxidation (Yang and Stockwell, 2015). Concurrently, adipose-derived pro-inflammatory cytokines (e.g., TNF-α and IL-6) upregulate hepatic hepcidin via the JAK2/STAT3 pathway, suppressing FPN-mediated iron export and causing intracellular iron overload (Salaye et al., 2019).

The second hit arises from mitochondrial dysfunction in steatotic hepatocytes, where impaired β-oxidation leads to ROS overproduction via Fenton reactions, synergizing with ACSL4-mediated lipid peroxidation to drive ferroptosis. Excess FFAs impair mitochondrial β-oxidation, leading to electron transport chain leakage and superoxide overproduction (Bessone et al., 2019). Superoxide reacts with labile iron via the Fenton reaction, generating hydroxyl radicals that initiate ACSL4-dependent peroxidation of PUFA-phospholipids (PUFA-PLs) (Conrad and Pratt, 2019). This process is amplified by downregulation of GPX4 and FSP1 in MAFLD:GPX4 inhibition: Obesity-induced hyperinsulinemia activates mTORC1, which suppresses NRF2-mediated GPX4 transcription (Sun et al., 2015a). FSP1 depletion: Adipocyte-secreted miR-27a-3p directly targets FSP1 mRNA, impairing CoQ10 regeneration and compromising membrane antioxidant defenses (Kraft et al., 2020).

Research shows that in methionine-choline deficient (MCD) diet-fed mice, hepatocyte-specific GPX4 knockout increased lipid ROS by 3.2-fold and accelerated fibrosis (p < 0.01 vs. wild-type), while ferrostatin-1 (5 mg/kg) reduced alanine aminotransferase (ALT) by 58% and hepatic MDA by 67% (Li et al., 2020). Human MAFLD biopsies show positive correlation between hepatic ACSL4 expression and histological activity index (r = 0.72, p = 0.003) (Tsurusaki et al., 2019). These findings establish ferroptosis as a central driver of MAFLD progression, linking adipose dysfunction to iron-redox imbalance in hepatocytes.

#### 3.5.2 Ferroptosis and metabolic-associated steatohepatitis (MASH)

In MASH, ferroptosis drives disease progression through a vicious cycle of iron metabolism imbalance - lipid peroxidation - inflammation cascade. Obesity - induced adipokine disorders (such as increased leptin) upregulate hepatic hepcidin through the JAK2/STAT3 signaling pathway, inhibiting FPN-mediated iron efflux and leading to the accumulation of free Fe^2+^ in the liver (Eder et al., 2020; Arfin et al., 2023). Meanwhile, the deficiency of ceruloplasmin prevents Fe^3+^ from binding to transferrin, further expanding the LIP (Kleven et al., 2018). Fe^2+^ generates ROS through the Fenton reaction, activates ACSL4, promotes the integration of AA into PL, and is oxidized by 15 - lipoxygenase (ALOX15) into toxic lipid peroxides (such as 4 - HNE) (Conrad and Pratt, 2019; Yang et al., 2014). This process is exacerbated by the collapse of the antioxidant defense system: obesity - related KEAP1 accumulation inhibits NRF2, resulting in a 60% decrease in the expression of GPX4 (p < 0.01), while adipocyte - derived exosomal miR - 34a degrades FSP1, weakening the antioxidant regeneration ability of CoQ10 (Doll et al., 2019; Sun et al., 2015a).

Ferroptosis and inflammation form a self - amplifying positive - feedback loop. Ferroptosis hepatocytes release damage - associated molecular patterns (DAMPs) such as HMGB1, which activates the TLR4/NF - κB pathway in Kupffer cells, triggering the secretion of IL - 1β and TNF - α (Tsurusaki et al., 2019). These inflammatory factors further inhibit the cystine transporter SLC7A11, deplete GSH, and upregulate ALOX15 through STAT6, forming a vicious cycle of “lipid peroxidation - inflammation” (Stockwell, 2022). Experimental evidence shows that hepatocyte - specific GPX4 knockout in mice fed with a MCD diet increases the fibrotic area by 3.5 - fold (p < 0.001), while the ferroptosis inhibitor ferrostatin - 1 can reverse this phenotype. In terms of clinical translation, the ACSL4 inhibitor rosiglitazone (4 mg/kg) reduces hepatic 4 - HNE by 72% in an arsenic - induced MASH model, and a phase II clinical trial (NCT04171740) has confirmed that sodium selenate (200 μg/day) normalizes ALT in 47% of patients (Choi et al., 2025). These findings establish targeting ferroptosis as a core strategy for the treatment of MASH.

#### 3.5.3 Ferroptosis and liver fibrosis

Ferroptosis plays a dual role in liver fibrosis by modulating HSC activation and extracellular matrix (ECM) deposition. Activated HSCs exhibit iron overload through upregulated TfR1 and NCOA4-mediated ferritinophagy, which releases Fe^2+^ via lysosomal degradation of ferritin. This iron fuels Fenton reactions, generating ROS that activate ACSL4 to drive AA-phospholipid peroxidation, triggering HSC ferroptosis (Muri et al., 2020). Clinically, TfR1 expression in fibrotic HSCs is 2.1-fold higher than in normal tissue (p < 0.001), correlating with collagen deposition (r = 0.69) (Reich et al., 2021). Regulatory networks further fine-tune this process: zinc finger protein 36 (ZFP36) suppresses ferroptosis by degrading ACSL4 mRNA, while ELAV-like RNA-binding protein 1 (ELAVL1) stabilizes BECN1 transcripts to promote autophagy-dependent ferroptosis. In CCl_4_-induced mice, ELAVL1 knockout reduced HSC ferroptosis by 58% and fibrosis area by 42% (p < 0.01) (Chen et al., 2022). Concurrently, magnesium isoglycyrrhizinate induces HSC ferroptosis via HO-1/p53 signaling, where HO-1-derived Fe^2+^ activates p53 to drive iron-mediated cell death—a process blocked by p53 inhibitors (Latorre et al., 2021).

The fibrotic microenvironment amplifies ferroptosis through Kupffer cell-derived IL-6/STAT3 signaling, which upregulates SLC39A14 (ZIP14) in HSCs to promote zinc influx and inhibit FPN-mediated iron export, exacerbating iron overload (Lu et al., 2022). Iron chelators like deferoxamine (DFO) reverse this cascade, reducing liver stiffness by 23% (p = 0.02) in a Phase II trial (NCT04842747). However, ferroptosis exhibits context-dependent duality: while HSC ferroptosis alleviates fibrosis, hepatocyte ferroptosis releases TGF-β1 and DAMPs that activate quiescent HSCs, creating a profibrotic loop. Thus, selectively targeting HSC ferroptosis—e.g., sorafenib-induced ZFP36 degradation—without harming hepatocytes is critical for therapeutic efficacy.

#### 3.5.4 Ferroptosis and obesity-related hepatocellular carcinoma (HCC)

Ferroptosis plays a dual role in obesity-related HCC, where metabolic dysfunction and chronic inflammation create a permissive microenvironment for both tumor progression and iron-dependent cell death. In obese individuals, NTBI accumulation, driven by adipose-derived hepcidin-mediated ferroportin suppression, synergizes with lipid peroxidation to heighten ferroptosis susceptibility (Verna Elizabeth et al., 2020). This is exacerbated by NRF2 pathway suppression due to hyperinsulinemia-induced KEAP1 stabilization, which downregulates GPX4 and depletes GSH, rendering HCC cells vulnerable to sorafenib-induced ferroptosis. Clinically, HCC patients with metabolic syndrome exhibit 40% lower GPX4 expression compared to non-metabolic counterparts (p < 0.001), correlating with reduced overall survival (Sun et al., 2015a). Paradoxically, although sorafenib-induced ferroptosis has demonstrated therapeutic potential in HCC (Bai et al., 2017), the overexpression of obesity-associated metallothionein 1G (MT-1G) can scavenge unstable Fe^2+^, thereby attenuating the drug’s efficacy. This dual role suggests the adoption of a context-dependent therapeutic strategy. Early-stage HCC, characterized by iron redox vulnerability, requires the promotion of ferroptosis to achieve therapeutic effects. Conversely, in late-stage HCC accompanied by MT-1G-driven drug resistance, inhibiting ferroptosis to prevent the clearance of unstable Fe^2+^ by MT-1G overexpression is necessary to ensure the efficacy of sorafenib (Zhu et al., 2020).

## 4 Ferroptosis-based pharmacotherapy strategy for obesity-related metabolic disease

**FIGURE 5:**
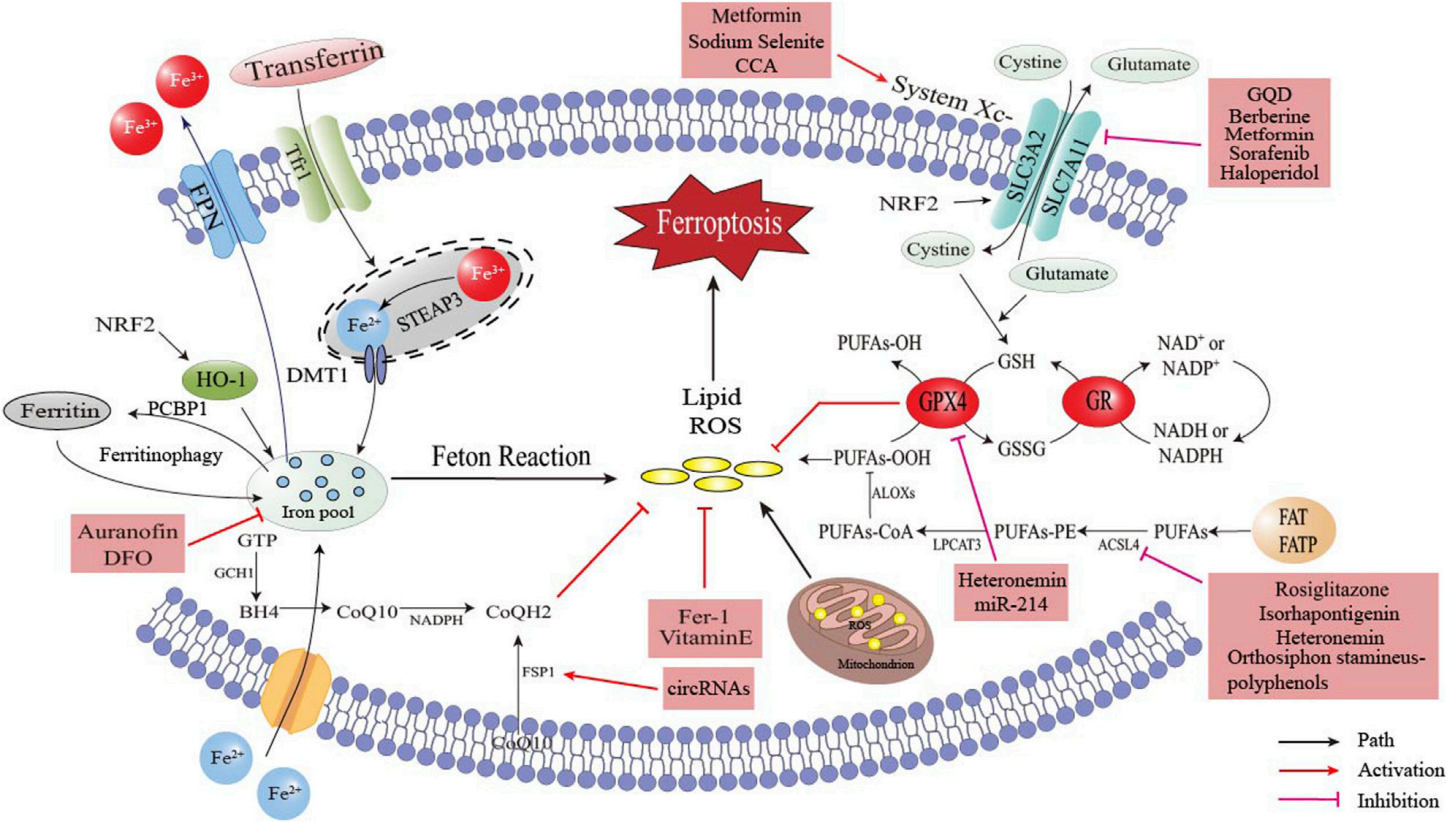
The occurrence and regulation of ferroptosis and the targets of related drugs.

Ferroptosis has been firmly convinced to be related to the occurrence and development of obesity-related chronic metabolic diseases. Consequently, designing corresponding and rational targeted drugs based on the regulatory pathways of ferroptosis holds great practical significance and promising clinical application prospects. As depicted in Figure 5, several active substances have been demonstrated to inhibit ferroptosis and ameliorate the symptoms of obesity-related chronic metabolic diseases by means of removing excess iron, neutralizing lipid peroxides and activating pathways such as the NRF2 signal. It is worth mentioning that Traditional Chinese medicine (TCM) has shown great potential in the research of ferroptosis. Researchers have identified a variety of Chinese herbs that can regulate ferroptosis. For example, Gu et al. (Gu et al., 2024) proposed that berberine could inhibit ferroptosis by activating the SLC7A11/GSH/GPX4 signaling pathway, and ultimately remarkably improve the bone loss induced by MAFLD; Ding et al. (Ding et al., 2023) discovered that epigallocatechin gallate (EGCG) could protect against high - fat - diet - induced hepatic lipid toxicity by inhibiting hepatic ferroptosis; Luo Q et al. (Luo et al., 2025) demonstrated both *in vitro* and *in vivo* in the context of MAFLD that Gegen Qinlian Decoction (GQD) could ameliorate MAFLD by suppressing ferroptosis, and its mechanism of action was likely associated with the regulation of the Nrf2/SLC7A11/GPX4 signaling pathway; The research findings of Zhang X et al. (Zhang et al., 2025) indicated that GQD could improve the liver injury in type 2 diabetes mellitus (T2DM) by regulating Nrf2 to inhibit ferroptosis.

As shown in Table 1, we classified ferroptosis - targeted therapies according to the mechanisms of action of the drugs. These classifications are supported by pre - clinical and clinical evidence. Despite promising preclinical data, clinical translation faces challenges. For instance, deferoxamine, a representative drug of iron homeostasis modulators, caused ocular toxicity in trials (NCT02502773, Phase I/II), while FSP1 inhibitors lack specificity. Future research should prioritize tissue-targeted delivery systems, such as iron death modulators encapsulated in nanoparticles (Gotardo et al., 2023). Ferrostatin-1, a representative lipid peroxidation inhibitor, demonstrated dose-dependent renal tubular injury in rodent models (preclinical) (Miotto et al., 2020) (Carpellini et al., 2023). Due to its rapid oxidative inactivation and a plasma half-life of only 1.2 h, prodrug design is necessary to extend its half-life. High-dose administration of Cryptochlorogenic Acid (CCA), a representative GPX4 pathway enhancer, can lead to intestinal flora imbalance (NCT03928821, Phase I), suggesting the formation of complexes to improve intestinal absorption (Zhou, 2020). Isorhapontigenin, one of the representative drugs of ACSL4/LPCAT3 inhibitors (NCT04892749, Phase I/II), has an oral bioavailability of only 8.7% due to glucuronidation. Exosome delivery could be considered to improve bioavailability (Chen et al., 2022). Representative Ferroptosis Sensitizers include Sorafenib + Haloperidol, which reduced tumor volume by 68% (p < 0.001) in obese patients with HCC (n = 15), but with a 15% incidence of QT interval prolongation (NCT04904419, Phase Ib) (Bai et al., 2017) (Zhu et al., 2020). This article discusses the potential side effects, bioavailability issues, and alternative drug delivery strategies of five different mechanisms of ferroptosis-targeted drugs by analyzing their clinical research status. It also highlights the limitations of current drug treatments and innovative directions for future targeted drug development, providing new avenues for the prevention and treatment of obesity-related metabolic diseases.

**TABLE 1:** Ferroptosis - targeted drugs are classified according to their mechanis

Category	Representative drug	Mechanism of action	Preclinical or clinical evidence support
Iron Homeostasis Modulators	Auranofin	Activates the NF-κB/IL-6/JAK-STAT pathway to upregulate hepcidin	Reduce systemic iron overload and hepatic iron apoptosis in MAFLD (Rasmussen et al., 2021)
	Deferoxamine (DFO)	Chelates excess iron, alleviating lipid peroxidation in diabetic nephropathy	Alleviates lipid peroxidation in diabetic nephropathy/Reduces liver stiffness in patients with fibrosis (Gotardo et al., 2023)
	Metformin	Suppresses p53-mediated SLC7A11 downregulation	Recovery of glutathione synthesis in hyperlipidemia models (Ma W.-Q. et al., 2021)
Lipid Peroxidation Inhibitors	Ferrostatin-1	Scavenges lipid ROS	Reversal of CCl_4_ induced liver damage (Miotto et al., 2020) (Carpellini et al., 2023)
	Vitamin E (α-tocopherol)	Reduces ALT levels	ALT levels in MASH patients were reduced by 43% (Schwabe et al., 2020)
	Orthosiphon stamineus polyphenols	Downregulate ACSL4 and upregulate FTH1	Inhibited renal ferroptosis in diabetic nephropathy (Ji et al., 2023) (Lei et al., 2024)
GPX4 Pathway Enhancers	Sodium Selenite	Activates GPX4	Reduce MASH severity by 50% in MCD diet models (Qi et al., 2020)
	Cryptochlorogenic Acid (CCA)	Activates the XC-/GPX4/NRF2 axis	Inhibiting pancreatic ferroptosis in diabetic rats (Zhou, 2020)
	Metformin	Enhances NRF2-mediated GPX4 transcription	Decreased liver MDA by 40% (Bai et al., 2017)
ACSL4/LPCAT3 Inhibitors	Rosiglitazone	A PPARγ agonist that suppresses ACSL4	Reduced liver 4-HNE by 72% in arsenic-induced MASH (Wei et al., 2020) (Tao et al., 2024)
	Isorhapontigenin	Inhibits mitochondrial ACSL4 via the PRDX2-MFN2 axis	Prevention of diabetic cardiomyopathy (Chen et al., 2022)
	Heteronemin	A marine terpenoid that downregulates ACSL4 and GPX4	Induced hepatocellular carcinoma ferroptosis (Chang et al., 2021)
Ferroptosis Sensitizers	Sorafenib + Haloperidol	Haloperidol inhibits sigma-1 receptor-mediated NRF2 activation	The volume of HCC tumor in obese mice was reduced by 68% (Bai et al., 2017) (Zhu et al., 2020)
	CircRNAs	Over 20 circRNAs suppress ferroptosis via FSP1/CoQ10 or system Xc- modulation	It shows promise in a preclinical HCC model (Arabpour et al., 2024) (Liang et al., 2024)
	miR-214	Targets cystathionine β-synthase (CBS), depleting GSH	The HCC cells were sensitized to iron apoptosis induced by erastin (Wang et al., 2024)

## 5 Current research gaps

### 5.1 Gender and age-dependent differences in ferroptosis susceptibility

There are significant gender and age differences in the incidence and clinical manifestations of metabolic diseases, but the mechanisms underlying their role in ferroptosis regulation remain unclear. Epidemiological studies have shown a significant increase in the prevalence of T2DM and MAFLD among postmenopausal women, while obese men are more likely to progress to metabolic-associated steatohepatitis (MASH). Sex hormones may affect ferroptosis susceptibility by regulating iron metabolism pathways: estrogen can upregulate the expression of hepcidin in the liver, inhibiting intestinal iron absorption and promoting iron storage in macrophages, while testosterone reduces hepcidin levels by activating androgen receptors (Cheung and Cheng, 2016). Age-related imbalances in iron homeostasis may also reshape ferroptosis sensitivity: mitochondrial dysfunction in hepatocytes of elderly individuals leads to iron overload, and the degradation of the antioxidant defense system (such as reduced glutathione synthesis) may exacerbate ferroptosis damage. However, there is currently a lack of comparative studies on the expression profiles of key ferroptosis molecules (such as GPX4 and ACSL4) in adipose tissue and liver across different genders and age groups.

### 5.2 Lack of clinical validation of ferroptosis biomarkers in human obesity

Although ferroptosis assessment indicators have been established in animal models, including lipid peroxidation products (such as 4-HNE), iron ion concentration, and key gene expression, specific biomarkers for human obesity patients remain elusive (Huang et al., 2023). Clinical biopsy results show a positive correlation between lipid peroxidation deposition and the expression of the ferroptosis core gene ACSL4 in the liver tissue of patients with obesity-related fatty liver disease (Stockwell et al., 2017). However, its quantitative association with disease progression (such as liver fibrosis staging) is not yet clear. Regarding blood markers, circulating hepcidin and soluble transferrin receptor (sTfR) can reflect systemic iron load but cannot accurately identify tissue-specific ferroptosis events. Additionally, the clinical detection value of antioxidant indicators such as glutathione (GSH)/oxidized glutathione (GSSG) ratio and coenzyme Q10 levels in the obese population still requires validation through large sample cohort studies (Xu et al., 2023).

### 5.3 Controversy over the bidirectional regulatory mechanism of HO-1 in ferroptosis

Heme oxygenase-1 (HO-1), as a core node in the ferroptosis regulatory network, exhibits significant environment-dependent contradictory evidence for its function. According to classical theory, HO-1 exerts a protective effect by degrading heme to generate antioxidant products (carbon monoxide, bilirubin). However, recent studies have found that the free iron catalyzed by HO-1 can promote lipid peroxidation through the Fenton reaction, thereby exacerbating ferroptosis. In metabolism-related hepatocellular carcinoma (HCC), high-fat diet-induced HO-1 overexpression can promote tumor cell ferroptosis through iron ion release, while HO-1 can also reduce liver damage by inhibiting the NF-κB pathway in a chronic inflammatory environment (Luo et al., 2022; Zheng et al., 2023). The dynamic changes of this bidirectional effect during MAFLD progression are not yet elucidated: in the early stages of liver injury, HO-1 may delay disease progression through antioxidant effects; however, under iron overload conditions, its role in promoting iron ion release may accelerate hepatocyte death, forming a vicious cycle. There is an urgent need to analyze the spatio-temporal specific regulatory mechanism of HO-1 on ferroptosis in different cell types (such as hepatocytes and Kupffer cells) through conditional gene knockout models (Yang et al., 2023; Malaguarnera et al., 2005).

### 5.4 Bottlenecks in the delivery system for tissue-specific targeted therapy

The cell type specificity of ferroptosis determines the complexity of therapeutic intervention. Taking the liver as an example, excessive activation of hepatocyte ferroptosis can lead to liver injury, while inhibition of ferroptosis in hepatic stellate cells (HSCs) may promote fibrosis progression. Preclinical studies have shown that sulfasalazine targeting System Xc^−^ can induce ferroptosis in liver cancer cells, but there are differences in ferroptosis sensitivity among HSCs (Scott, 2008; Marc et al., 2020). Additionally, the insufficient liver targeting of ferroptosis inducers (such as sorafenib) may trigger off-target toxicity in organs such as the kidneys and heart. Developing tissue-specific delivery systems based on nanoparticles (such as liposome carriers targeting the asialoglycoprotein receptor of hepatocytes), combined with microenvironment-responsive release mechanisms (such as acid-sensitive polymers), will be a key strategy to address the narrow therapeutic window issue (Fan et al., 2019; Gaul et al., 2020).

## 6 Conclusion and prospects

Obesity stands as a profound risk factor for chronic metabolic diseases. In obese individuals, excessive fat accumulation can impact the body’s glucose and lipid metabolism. Overall, obesity significantly increases the incidence of chronic metabolic diseases during development, posing a serious threat to people’s health. To date, there is no universally effective strategy to treat obesity-related metabolic disorders. Ferroptosis, a novel form of cell death, has been identified as playing a crucial role in the pathogenesis of obesity-associated chronic metabolic diseases. However, several key scientific questions remain to be deeply explored. Current research gaps include the lack of biomarkers for ferroptosis in human obesity, insufficient data on gender-specific differences, the dual regulatory role of ferroptosis, and bottlenecks in tissue-specific targeted therapy delivery systems. This article comprehensively reviews the role, pathophysiology, prevention, and treatment strategies of ferroptosis in obesity-related chronic metabolic diseases. It points out directions for basic research on ferroptosis, raises urgent needs for developing precise intervention strategies, and provides new insights into the treatment and study of obesity-related chronic metabolic diseases in the future. More comprehensive and in-depth research will be conducted in the field of endocrinology and metabolism to overcome obesity-related chronic metabolic diseases and achieve ferroptosis-based therapies.

## Glossary

AA: arachidonic acid
AACE: the American association of clinical endocrinologists
ACE: the American endocrine society
ACSL4: Acyl-CoA synthetase long-chain family member 4
ALT: alanine aminotransferase
AMPK: AMP-activated Protein Kinase
BAP1: brca1-associated protein 1
BH4: tetrahydrobiopterin
CBS: cystathionine β-synthase
CCA: cryptochlorogenic Acid
CP: ceruloplasmin
CoQ: coenzyme Q
CoQ10: coenzyme Q10
DAMPs: damage - associated molecular patterns
DHFR: dihydrofolate reductase
DMT1: divalent metal transporter 1
DPI: diphenyl iodonium iodide
DPP4: dipeptidyl peptidase 4
ECM: extracellular matrix
ELAVL1: ELAV-like RNA-binding protein 1
FFA: free fatty acids
FINs: ferroptosis-inducing compounds
FPN: ferroportin
FSP1: ferroptosis suppressor protein 1
FTH1: ferritin heavy chain 1
GSH: glutathione
GCH1: GTP cyclohydrolase 1
GPX4: glutathione peroxidase 4
GQD: Gegen Qinlian Decoction
HCC: hepatocellular carcinoma
HFD: high fat diet
HO-1: heme oxygenase-1
HSC: hepatic stellate cells
HSF1: heat shock factor 1
HSPB1: HSP-binding factor 1
IR: insulin resistance
LIP: labile iron pool
LOX: lipoxygenase
LPCAT3: Lys phosphatidylcholine acyltransferase 3
MCD: methionine and choline deficient diet
MDM2: murine double minute 2
MT-1G: metallothionein-1G
NADPH: nicotinamide adenine dinucleotide phosphate
NADH: nicotinamide adenine dinucleotide
MAFLD: metabolic-associated fatty liver disease
MASH: non-alcoholic steatohepatitis
NCOA4: nuclear receptor coactivator 4
NQO1: NADPH quinone oxidoreductase 1
NRF2: nuclear factor erythroid 2-related factor 2
NTBI: non-transferrin-bound iron
PCOS: polycystic ovary syndrome
PCBP1: poly (rC) binding protein 1
PL: phospholipids
PLPO: phospholipid peroxidation
PUFA: polyunsaturated fatty acid
PUFA-PL: polyunsaturated fatty acyl moieties in phospholipids
ROS: reactive oxygen species
SLC7A11/xCT: recombinant solute carrier family 7, member 11
SLC3A2: recombinant solute carrier family 3, Member 2
TBI: transferrin-bound iron
Tfr1: transferrin receptor 1
WAT: white adipose tissue
ZFP36: zinc finger protein 36
